# Hydrogen Peroxide Variation Patterns as Abiotic Stress Responses of *Egeria densa*

**DOI:** 10.3389/fpls.2022.855477

**Published:** 2022-05-16

**Authors:** Takashi Asaeda, Mizanur Rahman, Xia Liping, Jonas Schoelynck

**Affiliations:** ^1^Hydro Technology Institute Co, Ltd., Tokyo, Japan; ^2^Research and Development Center, Ibaraki, Japan; ^3^Department of Environmental Science, Saitama University, Saitama, Japan; ^4^Department of Biology, University of Antwerp, Wilrijk, Belgium

**Keywords:** abiotic stress, hydrogen peroxide, stress responses, stress indicator, macrophyte management, *Egeria densa*, photoinhibition, iron stress

## Abstract

In vegetation management, understanding the condition of submerged plants is usually based on long-term growth monitoring. Reactive oxygen species (ROS) accumulate in organelles under environmental stress and are highly likely to be indicators of a plant’s condition. However, this depends on the period of exposure to environmental stress, as environmental conditions are always changing in nature. Hydrogen peroxide (H_2_O_2_) is the most common ROS in organelles. The responses of submerged macrophytes, *Egeria densa*, to high light and iron (Fe) stressors were investigated by both laboratory experiments and natural river observation. Plants were incubated with combinations of 30–200 μmol m^–2^ s^–1^ of photosynthetically active radiation (PAR) intensity and 0–10 mg L^–1^ Fe concentration in the media. We have measured H_2_O_2_, photosynthetic pigment concentrations, chlorophyll *a* (Chl-a), chlorophyll *b* (Chl-b), carotenoid (CAR), Indole-3-acetic acid (IAA) concentrations of leaf tissues, the antioxidant activity of catalase (CAT), ascorbic peroxidase (APX), peroxidase (POD), the maximal quantum yield of PSII (F_v_ F_m_^–1^), and the shoot growth rate (SGR). The H_2_O_2_ concentration gradually increased with Fe concentration in the media, except at very low concentrations and at an increased PAR intensity. However, with extremely high PAR or Fe concentrations, first the chlorophyll contents and then the H_2_O_2_ concentration prominently declined, followed by SGR, the maximal quantum yield of PSII (F_v_ F_m_^–1^), and antioxidant activities. With an increasing Fe concentration in the substrate, the CAT and APX antioxidant levels decreased, which led to an increase in H_2_O_2_ accumulation in the plant tissues. Moreover, increased POD activity was proportionate to H_2_O_2_ accumulation, suggesting the low-Fe independent nature of POD. Diurnally, H_2_O_2_ concentration varies following the PAR variation. However, the CAT and APX antioxidant activities were delayed, which increased the H_2_O_2_ concentration level in the afternoon compared with the level in morning for the same PAR intensities. Similar trends were also obtained for the natural river samples where relatively low light intensity was preferable for growth. Together with our previous findings on macrophyte stress responses, these results indicate that H_2_O_2_ concentration is a good indicator of environmental stressors and could be used instead of long-term growth monitoring in macrophyte management.

## Introduction

Submerged macrophytes are exposed to various abiotic and biotic stressors in their natural environment. Flow rates, metal ion concentrations, water temperature, light conditions, eutrophication, allelopathy, and pathogens are common environmental stressors. Recent studies have suggested that salinity (Khalid et al., 2020, 2021), conditions in flume facilities of submerged freshwater macrophytes (Vettori and Rice, 2019) can also play a major role in plant environmental stress. Plant cells exhibit a variety of responses to radicals, depending on their intracellular level. ROS levels ranging from 0.02 to 0.05 μM are involved in a normal signal transduction mechanism and could be beneficial with different Fe and light conditions (Kovalchuk, 2010). The accretion of reactive oxygen species (ROS) is prevented by antioxidant activities under usual conditions (Caverzan et al., 2012). However, under environmental stress, the ROS levels overcome the defense mechanism and create oxidative stress in plants (Gill and Tuteja, 2010). Although ROS are the essential byproducts of photosynthesis, an excess amount of solar energy generates ROS superoxide radicals, affecting the photosystem II (PSII) (Pospíšil, 2016). These superoxide radicals are catalyzed by superoxide dismutase (SOD), generating H_2_O_2_ (Prasad et al., 1994, 2015; Asada, 1999).

Under abiotic or biotic stress, plants generate physiological responses, including the accumulation of ROS in the organelles of their cells. The accumulation of ROS is harmful to plants; its presence in cells and tissues can cause oxidative stress, which denatures proteins, lipids, and DNA. ROS are also important for growth regulation and signaling mechanisms (Gapper and Dolan, 2006; Einset et al., 2007; Shetty et al., 2008; Salleh et al., 2016).

Macrophytes are sensitive to even minor changes in light intensity (Rae et al., 2001; Imamoto et al., 2008). As submerged macrophytes are usually exposed to relatively weak light, even moderate light in terrestrial areas may be a stress source for them. Therefore, the water depth, which is strongly correlated with the incident light intensity, is an important factor for the growth of macrophytes (Liu et al., 2018; Asaeda et al., 2020). Further, plants—including submerged macrophytes—have a defense capacity against stress, depending on their physiological status. Thus, plants under various light conditions, including variational characteristics, may have different capacities to respond to stressors.

**FIGURE 1:**
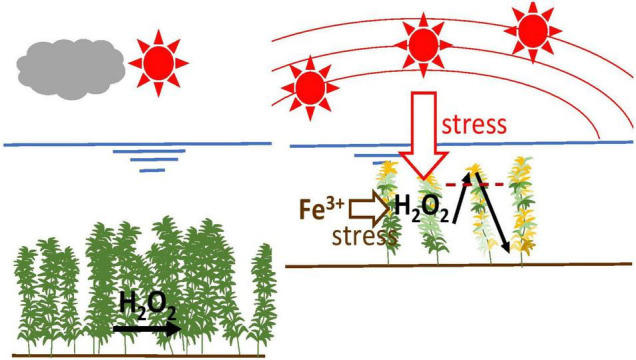
Overall overview of stress mechanism based on light intensity and Fe.

Fe is an essential element, particularly as it is important for the electron transport chain in photosynthesis as well as in antioxidant enzymes (Rabotti et al., 1995; Becana et al., 1998; Connolly and Guerinot, 2002). However, the excessive presence of Fe in the environment is toxic and is one of the major oxidative stress sources for plants. Because of its strong reactivity with oxygen, Fe is a difficult element for aerobic organisms to handle. Fe can catalytically promote the generation of hydroxyl radicals through Fenton’s reaction (Graf et al., 1984; Pinto et al., 2003; Halliwell, 2006). Thus, both high and low concentrations of Fe cause problems in plants, including submerged macrophytes (Bakker et al., 2016). The Figure 1 shows an overview of stress mechanism based on light intensity and Fe.

In plant management, the condition of plants is investigated mainly by the long-term casual observation and monitoring of growth parameters after treatment. It normally takes several months or years to identify the difference in the vegetation condition compared with the target condition. The longer it takes to recognize this shift, the more costly it becomes to reform the management plan. If a method could be applied to measure the plant’s condition directly by physiological factors, this time-consuming process in plant management could be avoided.

Among ROS, the accumulation of H_2_O_2_ is being widely adopted as a stress indicator in various plant stress studies, such as trees, shrubs, crops, and macrophytes (Kuźniak and Urbanek, 2000; Mittler, 2002; Suzuki et al., 2014; Zhang et al., 2014; Niu and Liao, 2016; Parveen et al., 2019; Asaeda et al., 2020). Compared with other ROS, H_2_O_2_ is relatively stable (Sharma et al., 2012) and can be quantified chemically with minimum losses (Satterfield and Bonnell, 1955). Therefore, the H_2_O_2_ concentration in the plant tissues was considered in the present research in order to review the possibility of H_2_O_2_ concentration as an indicator of stress intensity in vegetation management.

Our previous studies on abiotic stress (turbulence, heat, hypoxia, H_2_S) on aquatic macrophytes revealed that each stressor in the their habitat’s normal condition has the tendency to enhance the plant’s H_2_O_2_ concentration proportionately to the stress intensity (Ellawala et al., 2011; De Silva and Asaeda, 2017a,b; Parveen et al., 2017). With a combination of stressors, the H_2_O_2_ accumulated for each stressor is estimated as the sum of each component and allows one to estimate the stress level compared with the threshold H_2_O_2_ level. The H_2_O_2_ contents have a high correlation to the chlorophyll contents, the growth rate, and the colony formation; thus, it is possible to estimate the stress level to make colonies based on the H_2_O_2_ concentration (Asaeda et al., 2020, 2021). It is extremely beneficial if we can evaluate the effect of different stressors by a single parameter. However, a low H_2_O_2_ concentration was also observed under an excessively high intensity of stresses present for a long period (Asaeda et al., 2020). Particularly in the field observation, stress intensity frequently changes, and it is difficult to evaluate the equivalent intensities from the different exposure period to the stressors and the experienced intensities.

In the present study, therefore, the variational trend of H_2_O_2_ and photosynthetic pigments was investigated, depending on the exposure period to single or combined stressors, with the aim of discovering the proper management procedure of macrophytes.

## Experimental Design

### Laboratory Experiments

A healthy stock of *Egeria densa* was collected from the Moto-Arakawa River in southern Saitama, Japan. The collected plants were cleaned with water to remove debris, and the attached algae were carefully separated by tweezers. Then, the plants were cultured in several glass tanks under laboratory conditions (25 ± 2°C, 12/12 h. photoperiod, PAR intensity 100–150 μmol m^–2^ s^–1^ using fluorescent lighting) for several months. Commercial sand (D_50_ < 0.2 mm) was used as a substrate, and a 5% Hoagland solution was provided as the nutrient media. Algae-free stocks were selected for the experiments.

The experiments were conducted by growing *E. densa* cuttings (7 cm long) in 500-mL narrow glass beakers (13.6 cm height × 7.5 cm outside diameter) without a substrate. Each beaker was wrapped with a reflective sheet so every part of plant tissue was homogeneously exposed to the same intensity of light. Two cuttings were firmly attached to a sponge and fixed to the bottom of each beaker. A Hoagland solution (5%) was provided as the nutrient source. Three light intensities (30, 100, and 200 μmol m^–2^ s^–1^ PAR intensity) and six concentrations of Fe (0, 0.5, 3, 5, 7, and 10 mg L^–1^) were chosen in order for the experiment to range from natural to extreme conditions. The light was provided by LED straight lights (Model LT-NLD85L-HN; OHM Electric Inc., Japan) with a 12/12 h photoperiod. The Fe concentration in the media was adjusted by adding the required FeCl_3_ amount to the tank. The control condition was maintained by keeping the plants in a 5% Hoagland solution (0.13 mg L^–1^ Fe) without any further treatment. Stress assays were performed after three or seven days of exposure.

Several sets of additional conditions were added to obtain the different patterns of solar radiation.

With 5% Hoagland media at 25 ± 2 or 20 ± 2°C, *E. densa* was grown in 30 × 18 × 20 cm reflective sheet-wrapped glass tanks under four PAR densities of 50, 100, 200, and 300 μmol m^–2^ s^–1^. In this set of experiments, analyses of the samples were conducted every 5 days for 30 days in order to observe the transition from the start of the experiment.

In another set of experiments, the *E. densa*-grown tanks were located outdoors and exposed to solar radiation for three consecutive clear days. Three tanks were prepared for each of either 20 ± 2 or 30 ± 2°C water temperature for the replicates. On the third day, every 3 h., from 6 am (just after sunrise) to 6 pm (slightly before sunset), samples were taken from the three tanks of each temperature and were subjected to analyses. The solar radiation intensity was measured at each sampling time.

### Field Sampling

Field sampling of *E. densa* was conducted in western Japan’s Saba River, midstream. Sampling was conducted on clear days with less than 20% cloud cover in August 2018. Plant conditions at river reaches were surveyed beforehand. Five to ten representative sites of healthy mono species communities of *E. densa* were selected from nearly stagnant water (less than 5 cm s^–1^ mean velocity) upstream of weirs.

Light intensity was measured from the surface to the bottom of the river at 10cm intervals.

Then, the canopy top shoots were carefully sampled, tightly sealed in plastic bags, and stocked in a frozen storage box with dry ice until they were brought to the laboratory for chemical analysis (H_2_O_2_, Chl-a, Chl-b, and CAR). The comparison with non-frozen samples indicated that the freezing process did not have any effect on the chemical composition. The water depth of the sites ranged from 0.25 to 1.0 m. The water quality parameters were within the common range for the area: temperature, 20–25°C; pH, 6.8–7.0; dissolved oxygen, 9.0 mg L^−1^; salinity, 0 ppt; turbidity, 0–35 NTU; and electrical conductivity, 5–11 ms m^–1^.

### Chemical Analyses

Plant lengths were measured using a millimeter scale at 5–7-day intervals. The SGR was calculated as the difference in the shoot length between two observations. The SGR was obtained by dividing the length by the duration and was expressed in cm day^–1^. At the end of the experiment, the plants were oven-dried at 70°C for 72 h. The dry weight (DW) of the shoots was measured to confirm the reliability of the shoot length as a reference parameter of the growth rate. The weight length^−1^ ratio was 4.0 ± 1.0 mg DW cm^–1^, regardless of conditions, except for the dying samples; thus, SGR values were used as the reference growth rate (Ellawala et al., 2011).

The Chl-a, Chl-b, and total CAR contents were spectrophotometrically (UV Mini 1210; Shimadzu, Japan) determined by extracting pigments of N, N-dimethylformamide after keeping dark for 24 h. The results were expressed in fresh weight (FW) (Porra et al., 1989). The chlorophyll fluorescence parameters were measured by fluorescence imaging (FC 1000-H; Photon Systems Instruments, Czech Republic) with auto image segmentation. Initially, the plants were dark-adapted for 20 min, and the maximum quantum efficiency of PSII (F_v_ F_m_^–1^) was obtained.

Apart from IAA, the stress assay compounds H_2_O_2_, CAT, APX, and POD were extracted by grinding the freeze-dried (with liquid nitrogen) fresh plant sample (∼500 mg) with an ice-cold, pH 6.0, 50 mM phosphate buffer. Polyvinylpyrrolidone (PVP) was added to the extraction to mask the effect of phenolic compounds in the plant materials. The sample extraction for endogenous IAA was performed by following a similar procedure, but distilled water was used as the extraction media. Then, the extractions were centrifuged at 5,000 × *g* and 4°C for 15 min, and the supernatant was separated and incubated at −80°C for further analysis. In each treatment, the extractions were performed in triplicate. All the results were expressed in FW.

The H_2_O_2_ contents were determined colorimetrically following the TiSO_4_ method (Satterfield and Bonnell, 1955), with modifications. The reaction mixture contained 750 μL of enzyme extract and 2.5 mL of 1% TiSO_4_ in 20% H_2_SO_4_ (v/v), which was centrifuged at 5,000 × *g* and 20°C for 15 min. The optical absorption of the developed yellow color was measured spectrophotometrically at a wavelength of 410 nm. The H_2_O_2_ concentrations in the samples were determined using the prepared standard curve for known concentration series. The H_2_O_2_ contents were expressed in μmol g^–1^ FW.

The absorption at 410 nm includes the effect of other soluble compounds (Cheeseman, 2006; Queval et al., 2008). Thus, the H_2_O_2_ concentration was calculated from the slope of the standard curve obtained from the known H_2_O_2_ concentration, which was offset, derived by the intercept absorption rate with zero H_2_O_2_ concentration samples (Cheeseman, 2006). The results were compared with those of the e-FOX method (Queval et al., 2008), and a suitable correlation (*R*^2^ = 0.98) was obtained. The results were presented as μmol g^–1^ FW.

The CAT activity was measured as follows: 100 μL of 10 mM H_2_O_2_ and 2.0 mL of 100 mM potassium phosphate buffer (PH 7.0) were added to the cuvette before 500 μL of enzyme extract was added to initiate the reaction. The optical absorbance reduction at 240 nm was recorded every 10 s for 3 min. Finally, the CAT activity was obtained using an extinction coefficient of 40 mM^–1^ cm^–1^ (Aebi, 1984). The APX activity was determined as follows: the reaction mixture contained 100 μL of enzyme extract, 200 μL of 0.5 mM ascorbic acid in 50 mM potassium phosphate buffer (PH 7.0), and 2 mL of 50 mM potassium phosphate buffer (PH 7.0). The reaction was initiated by adding 60 μL of 1 mM H_2_O_2_. The decrease in absorbance at 290 nm was recorded every 10 s. The APX activity was calculated using an extinction coefficient of 2.8 mM^–1^ cm^–1^ (Nakano and Asada, 1981). The POD activity was spectrophotometrically measured based on the oxidation of guaiacol with the presence of H_2_O_2_. The reaction mixture contained 3.0 mL of pH 6.5 potassium phosphate buffer, 40 μL of 30 mM H_2_O_2_, and 50 μL of 0.2 M guaiacol. The reaction was initiated by the addition of 100 μL of crude enzyme extract, and the increase in absorbance at 420 nm was recorded every 10 s for 3 min. Then, the absorbance change rate and POD activity were calculated using an extinction coefficient of 26.6 mM^–1^ cm^–1^ (Goel et al., 2003).

The concentration of endogenous IAA was also determined using a prepared standard curve for known concentration series. The reaction mixture contained an aliquot of enzyme extract (1.00 mL) and 2.00 mL of modified Salkowski’s reagent (1.00 mL of 0.5 M FeCl_3_ in 50 mL of 35% perchloric acid) (Gordon and Weber, 1951). The resultant color intensity was measured as absorbance after a 1 h incubation period at 25°C at a wavelength of 530 nm, and the results were presented as μg g^–1^ FW.

Chlorophyll fluorescence was measured using a chlorophyll fluorescence imaging technique (FC 1000-H; Photon Systems Instruments, Czech Republic) with auto image segmentation. The Fv Fm^–1^ value became highest 20–30 min. after darkening (20 min. is sufficient; Hubbart et al., 2012). Thus, plant segments were dark-adapted for 20 min. before measurement. The maximum quantum efficiency of PSII photochemistry (Fv Fm^–1^) was calculated using the equation Fv Fm^–1^ = (Fm - Fo) Fm^–1^, where Fv, Fm, and Fo are the variable, maximum, and minimum fluorescence in the dark-adapted state, respectively.

The initial and final lengths of apical tips were measured using a ruler. The relative SGR was calculated with the formula SGR = (FL - IL) days^–1^, where FL is the final length and IL is the initial length.

### Statistical Analyses

The collected data were tested for normality with the Shapiro–Wilk test before the statistical analyses were performed. All results were presented as the mean ± SD of three replicates. The data were subjected to a one-way analysis of variance (ANOVA) with Tukey’s *post hoc* test for mean separation. The *t*-test was performed where necessary. Bivariate analysis was used and followed by Pearson’s correlation method to evaluate the relationship between parameters. Statistical analyses were performed with IBM SPSS V25.

## Results

**FIGURE 2:**
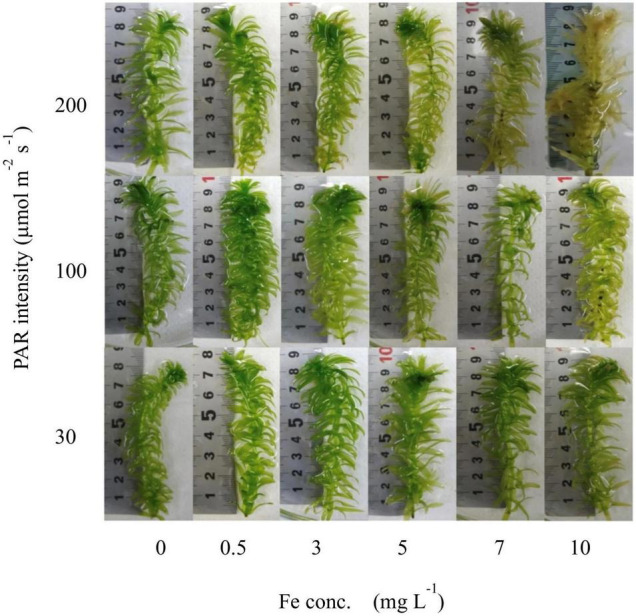
Visual conditions of plant shoots from the laboratory experiment after 7 days’ exposure to different light intensity and Fe conditions.

All samples from the laboratory experiments were in good condition at the end of the experiment, except for those exposed to an Fe concentration of 7–10 mg L^–1^ and a PAR intensity of 200 μmol m^–2^ s^–1^, which included samples that were almost dying. Therefore, these dying samples were excluded from the analyses. The growth media contained 0.13 mg L^–1^ of Fe by default, displayed as 0 mg L^–1^ in all figures for clarity, and other concentrations were scaled to it. With increasing Fe concentration and PAR intensity, the condition of the plants became worse. The conditions of the plants after 7 days are shown in Figure 2.

**FIGURE 3:**
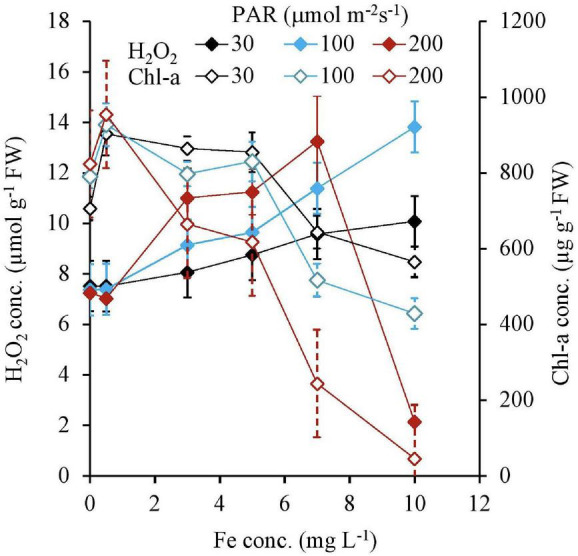
H_2_O_2_ and Chl-a concentrations of plant tissues after 7 days of the experiment as a function of Fe concentration in water and PAR intensities.

The H_2_O_2_ concentration and Chl-a concentration of tissues after 7 days are shown in Figure 3 as a function of Fe concentration. The H_2_O_2_ formation was the same regardless of PAR, with less than 0.5 mg L^–1^ Fe in the media. With the Fe concentration increasing from 0.5 mg L^–1^ Fe, the H_2_O_2_ concentration, which was higher with a higher PAR intensity, gradually increased for each PAR intensity group until reaching 7 mg L^–1^ of Fe concentration shown in Table 1. However, with 200 μmol m^–2^ s^–1^ of PAR, the H_2_O_2_ concentration suddenly declined at 10 mg L^–1^ Fe. The Chl-a concentration, which is low with higher light intensity, had a negative relation with the Fe concentration (Table 1).

**TABLE 1:** Relationship between Fe concentration and parameters for different light intensities.

Parameters	PAR (μmol m^–2^ s^–1^)	*t*	*R* ^2^	*p*
Chl-a	30	5.23	−0.934	0.006
	100	3.64	−0.878	0.022
	200	5.24	−0.934	0.006
H_2_O_2_ 0–7 mgL^–1^ Fe	30	11.5	0.982	0.00003
	100	10.3	0.979	0.00005
	200	8.18	0.937	0.0005

*P-values are obtained by one-way analysis of variance (ANOVA).*

**FIGURE 4:**
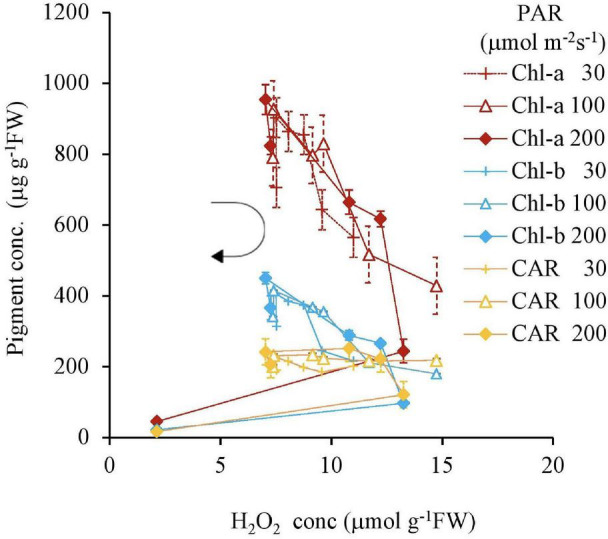
The relation between H_2_O_2_ and pigment concentrations of plant tissues after 7 days experiment as a function of Fe concentration in water and PAR intensities. The round arrow indicates the variational trend with increasing Fe concentration. Vertical bars indicate the standard deviation.

Both the Chl-a and Chl-b concentrations had high negative relationships with the H_2_O_2_ concentration, except for 0 and 10 mg L^–1^ Fe; however, they were not affected by the light intensity. In contrast, the CAR concentration did not have a high negative correlation with H_2_O_2_ (Table 2). All pigment concentrations significantly declined with 200 μmol m^–2^s^–1^ PAR and 10 mg L^–1^ Fe, which was associated with low H_2_O_2_ concentration (Figure 4).

**TABLE 2:** Relationship between H_2_O_2_ concentration and parameters for different light intensities.

Parameter	PAR (μmol m^–2^ s^–1^)	*t*	*R*	*p*
Chl-a	30	2.38	−0.765	0.05
	100	4.80	−0.923	0.002
	200	3.69	−0.905	0.014
Chl-b	30	5.62	−0.942	0.001
	100	6.40	−0.954	0.0007
	200	4.79	−0.940	0.0049
CAR	30	0.778	−0.363	0.466
	100	0.085	−0.043	0.935
	200	1.077	−0.528	0.330
Fv Fm^–1^	30	11.9	−0.986	2.1 × 10^–5^
	100	13.6	−0.989	9.7 × 10^–6^
	200	6.52	−0.966	0.0012
IAA	30	1.00	0.110	0.354
	100	0.443	0.448	0.674
	200	0.518	−0.216	0.626
SGR	30	0.758	0.354	0.477
	100	0.175	−0.087	0.868
	200	0.515	−0.285	0.628
CAT	30	17.8	−0.994	2 × 10^–6^
	100	4.66	−0.916	0.0038
	200	14.7	−0.993	2.6 × 10^–5^
POD	30	8.23	0.971	0.00017
	100	4.36	0.908	0.0047
	200	9.93	0.986	0.00018
APX	30	3.14	−0.848	0.019
	100	5.55	−0.941	0.0015
	200	10.87	−0.988	0.00011

*P-values are obtained by one-way analysis of variance (ANOVA), Bivariate analysis following Pearson’s correlation, and t-test.*

**FIGURE 5:**
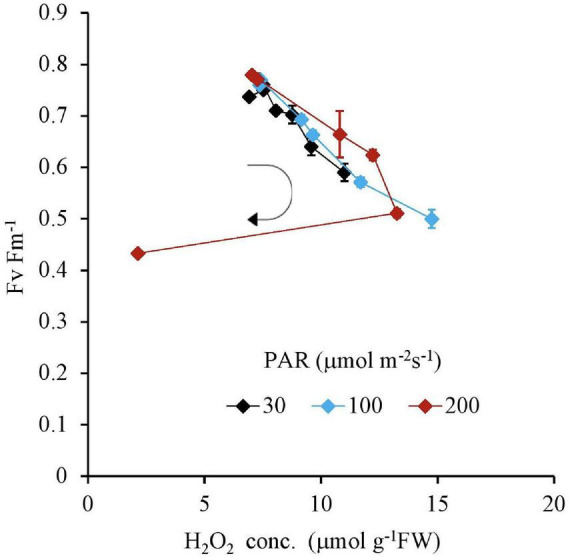
The relation between H_2_O_2_ and Fv Fm^– 1^ of plant tissues after 7 days as a function of Fe concentration in water and PAR intensities. The round arrow indicates the variational trend with increasing Fe concentration. Vertical bars indicate the standard deviation.

**FIGURE 6:**
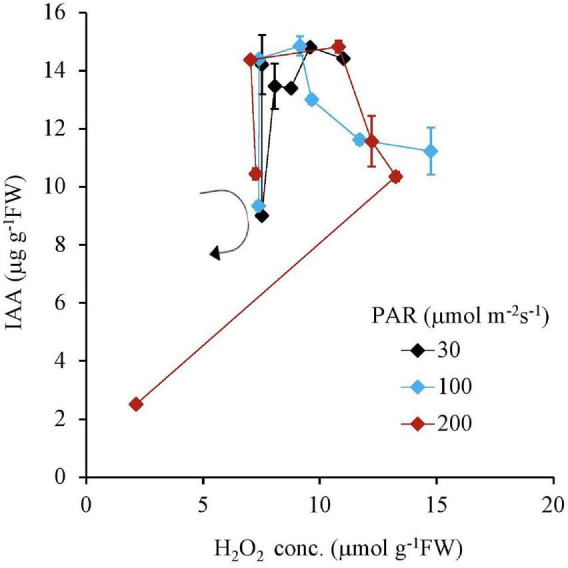
The relation between H_2_O_2_ and IAA concentrations of plant tissues after 7 days experiment as a function of Fe concentration in water and PAR intensities. The round arrow indicates the variational trend with increasing Fe concentration. Vertical bars indicate the standard deviation.

**FIGURE 7:**
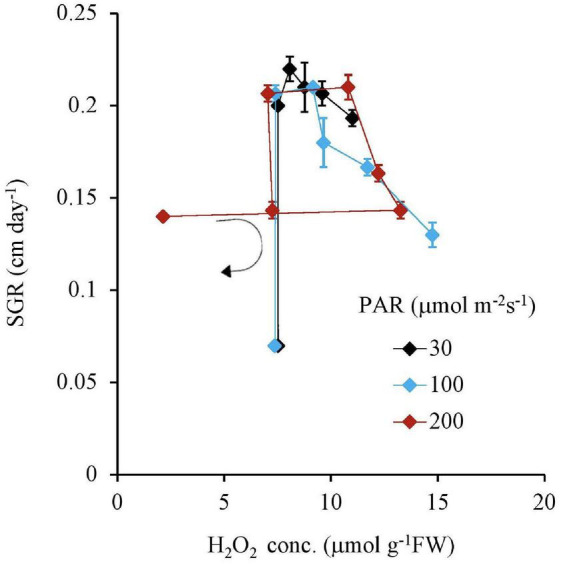
The relation between the H_2_O_2_ concentration of plant tissues and SGR after 7 days experiment as a function of Fe concentration in water and PAR intensities. The round arrow indicates the variational trend with increasing Fe concentration. Vertical bars indicate the standard deviation.

Similar to this trend, the Fv Fm^–1^ value had a unique negative correlation with the H_2_O_2_ concentration, except for the 200 μmol m^–2^s^–1^ of PAR and the 10 mg L^–1^ Fe condition (Table 2 and Figure 5). Both IAA and SGR had a similar relationship with the H_2_O_2_ concentration and had a significantly high correlation to each other, except for the 200 μmol m^–2^s^–1^ of PAR and the 10 mg L^–1^ Fe condition (Table 2 and Figures 6, 7).

**FIGURE 8:**
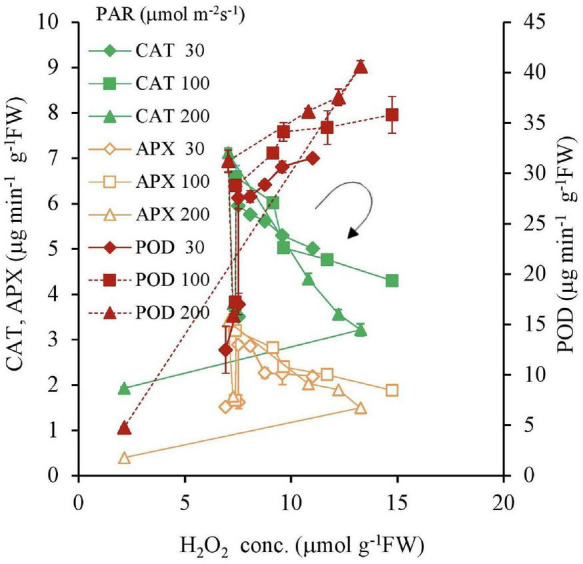
The relations between H_2_O_2_, CAT, POD, and APX concentrations of plant tissues after 7 days experiment as a function of Fe concentration in water and PAR intensities. The round arrow indicates the variational trend with increasing Fe concentration. Vertical bars indicate the standard deviation.

For 30, 100, and 200 μmol m^–2^s^–1^ of PAR, a negative correlation was found with an increasing Fe concentration for CAT and APX in Table 2 except for 0 mg L^–1^ Fe concentration and 10 mg L^–1^ and 200 μmol m^–2^ s^–1^ PAR; however, there was a positive correlation for POD (Table 2 and Figure 8).

### Long-Term Response to the Different Light Intensities

**FIGURE 9:**
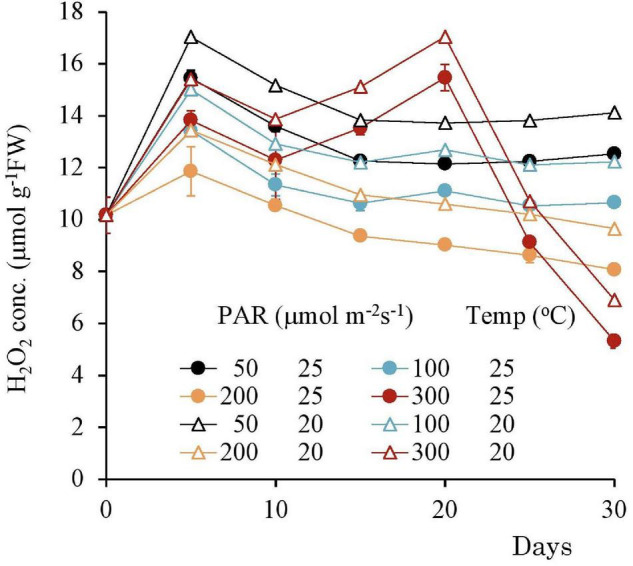
The variational trend of the H_2_O_2_ concentration of plant tissues after 7 days experiment with respect to different PAR. Vertical bars indicate the standard deviation.

**FIGURE 10:**
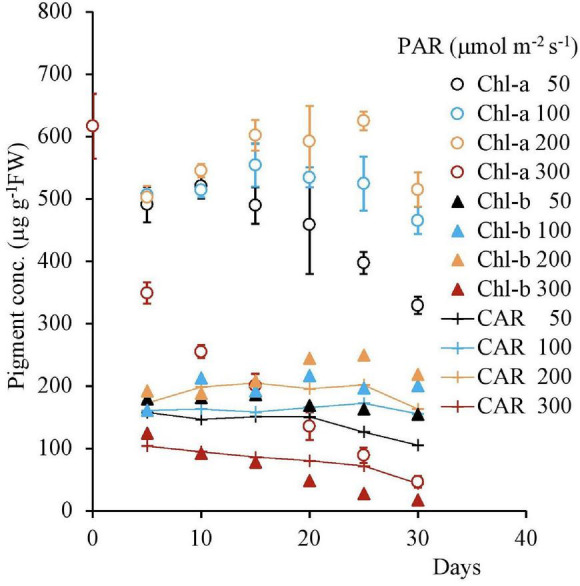
The variational trend of the pigment concentration of plant tissues with respect to various light intensities. Vertical bars indicate the standard deviation.

The H_2_O_2_ concentration variation from the beginning of the experiment to 30 days after its commencement shown in Figures 9, 10 with different light intensities. Regardless of temperature, the H_2_O_2_ concentration was higher with low PAR for 50–200 μmol m^–2^s^–1^ of PAR (Table 3). Although the H_2_O_2_ concentration rose after the experiments began, it declined afterward until the 15th day with 50–100 μmol m^–2^s^–1^ of PAR and then became stable; there was no significant correlation with time (*p* > 0.7 for 50–100 μmol m^–2^s^–1^ of PAR). However, the H_2_O_2_ concentration continued to decline slightly with 200 μmol m^–2^s^–1^, and the H_2_O_2_ concentration for 15–30 days had a significant negative correlation with PAR in 50–200 μmol m^–2^ s^–1^ [Table 3; empirically given by H_2_O_2_ (μmol g^–1^FW) = 0.025*PAR (μmol m^–2^s^–1^) + 14.9 for 20°C, and H_2_O_2_ (μmol g^–1^FW) = 0.025*PAR (μmol m^–2^s^–1^) + 11.1 for 25°C].

**TABLE 3:** Relationship between PAR and H_2_O_2_ concentration in 30 days experiment.

	PAR (μmol m^–2^ s^–1^)	Temperature (°C)	*t*	*R*	*p*
H_2_O_2_	50, 100, 200	20	5.70	−0.845	0.000078
H_2_O_2_	50, 100, 200	25	7.62	−0.905	0.000038

*P-values are obtained by t-test.*

In contrast, with 300 μmol m^–2^s^–1^, the H_2_O_2_ concentration tended to increase after 10 days until reaching 17 μmol g^–1^FW then, it significantly declined (Table 4).

**TABLE 4:** Relationship between parameters and the exposed period in 30 days experiment.

Parameter	PAR (μmol m^–2^ s^–1^)	Temperature (°C)	*t*	*R*	*p*
H_2_O_2_	50, 100, 200	20	2.51	−0.532	0.023
H_2_O_2_	50, 100, 200	25	2.51	−0.532	0.023
H_2_O_2_, Exposed period:5–20 days	300	20,25	3.21	0.849	0.032
H_2_O_2_, Exposed period 20–30 days	300	20,25	8.37	−0.973	0.0011
Chl-a	50	25	4.19	−0.902	0.014
	100		0.74	−0.347	0.499
	200		0.66	0.313	0.546
	300		14.6	−0.991	0.00012
Chl-b	50	25	3.32	−0.856	0.025
	100		1.04	0.461	0.893
	200		0.28	0.732	0.790
	300		6.34	−0.991	0.00011
CAR	50	25	3.51	−0.869	0.025
	100		0.143	0.0715	0.893
	200		0.285	−0.14	0.790
	300		6.34	−0.94	0.003
CAT	50	25	4.90	−0.926	0.008
	100		5.35	−0.937	0.006
	200		6.17	−0.951	0.004
	300		4.50	−0.914	0.011
POD	50	25	2.43	−0.772	0.072
	100		2.42	−0.770	0.073
	200		7.07	−0.962	0.002
	300		2.06	−0.717	0.002
APX	50	25	0.289	0.143	0.787
	100		2.59	−0.792	0.061
	200		2.39	−0.767	0.075
	300		4.24	−0.904	0.013

*P-values are obtained by one-way analysis of variance (ANOVA), Bivariate analysis following Pearson’s correlation, and t-test.*

The pigment concentration steadily declined after the beginning of the experiment, with 300 μmol m^–2^s^–1^ of PAR Shown in Table 4. However, with other PAR intensities, the pigment concentration did not indicate significant change. As the days progressed from 1 to 30, increasing PAR affected the pigment concentration, which in turn, gradually decreased the Chl-a, Chl-b, and CAR levels.

### Response to the Diurnal Variation of the Solar Radiation

**FIGURE 11:**
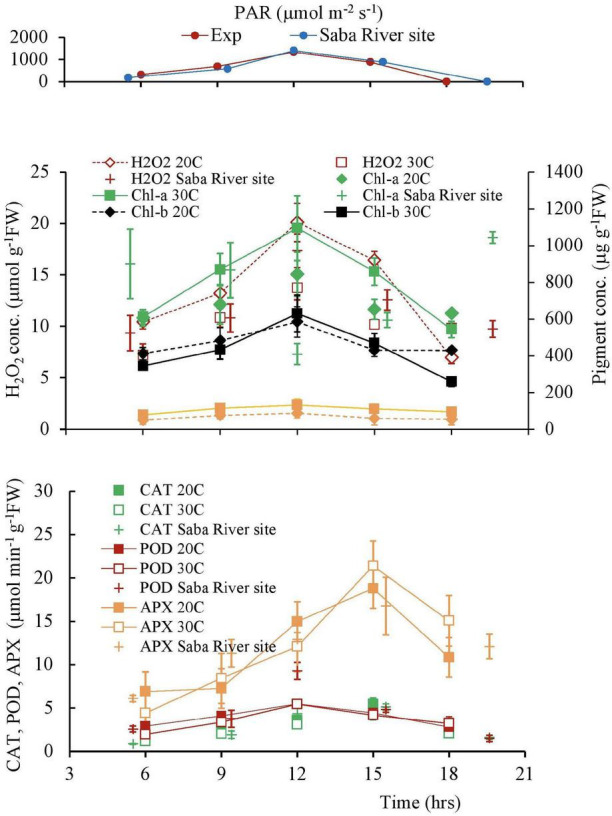
The variation patterns of the solar radiation, H_2_O_2_ concentration, and antioxidant activities of plant tissues in a day. Vertical bars indicate the standard deviation.

The diurnal variation is shown in Figure 10. Following the variation of PAR, the H_2_O_2_ concentration and Chlorophyll concentrations varied, increasing in the morning and decreasing in the afternoon. The H_2_O_2_ concentration varied in a single day at different temperatures. A similar trend is also observed in the Chlorophyll concentrations. However, compared with the symmetrical change in Chlorophyll, the H_2_O_2_ concentration was higher in the afternoon, compared with the morning changes (Figure 11 and Table 4).

The activities of CAT and POD are also shown in Figure 11. POD showed high fluctuation day time experiment compared with CAT. Although the POD activity followed the PAR and H_2_O_2_ variations, the CAT activity delayed approximately 3 h of the variational patterns of the PAR and H_2_O_2_ concentrations because CAT got the optimal concentration of H_2_O_2_ to react and give a maximum yield (Scandalios et al., 1997).

## Discussion

Apart from the present study, laboratory experiments were previously conducted to obtain the relationship between the H_2_O_2_ concentration and various types of stressors (Asaeda et al., 2017; Asaeda and Rashid, 2017; De Silva and Asaeda, 2017a; Parveen et al., 2017). These experiments showed a positive correlation between the H_2_O_2_ concentration and stress intensity. The present study clearly indicates that the accumulation and destruction processes of H_2_O_2_ and photosynthetic pigments in the tissues are positively or negatively proportionate to the stress intensity. Further, the presence of a combination of factors (Fe and light), which are common stressors, reveals the significant effects of two stressors on aquatic plants.

### Effect of Light on Stress Intensity

In the natural environment, an *E. densa* colony can be formed in lower light intensity, having a low level of H_2_O_2_ concentration within a few days. The threshold value for *E. densa* is 16 μmol g^−1^FW in the daytime (Asaeda et al., 2020), which is observed in high solar radiation and indicates plant tissue damage due to oxidative stress. It was observed that *E. densa* prefers a light intensity < 200 μmol m^–2^ s^–1^. When the light intensity is over 200 μmol m^–2^ s^–1^, plant tissues tend to show signs of H_2_O_2_ accumulation. High solar radiation disrupts plant metabolism, creating hypoxic conditions. As a result, *E. densa* plants deteriorate (Asaeda et al., 2020).

In the laboratory studies, it was confirmed that *E. densa* has a preferred PAR intensity ranging from 30 to 200 μmol m^–2^ s^–1^. This indicates the light tolerance of *E. densa* and its preferred environmental conditions.

The H_2_O_2_ concentration slightly declined and then stabilized when exposed to less than 200 μmol m^–2^s^–1^ of PAR intensity. However, with 300 μmol m^–2^s^–1^, H_2_O_2_ gradually increased to ∼14 μmolg^–1^FW of H_2_O_2_ concentration and then suddenly declined to the lower level. On the other hand, the Chl-a concentration steadily declined with 200 μmol m^–2^s^–1^ of PAR after the experiment began, although there was almost no effect with lower light intensity. Therefore, slightly less than 200 μmol m^–2^s^–1^ of PAR seems to be the optimal light intensity for this species.

In diurnal changes of solar radiation, H_2_O_2_ is higher in the afternoon compared with the morning for the same light intensity. Elevated detoxifying ROS activity is one of the prime strategies plants often possess in response to abiotic stress. Though the POD antioxidant activity nearly follows the variation of H_2_O_2_, the CAT and APX activities are delayed nearly 3 h (Tables 5, 6). The delay of scavenging activity seems to allow the high H_2_O_2_ in the afternoon.

**TABLE 5:** The relation of parameters with the diurnal solar radiation.

Parameter	Temperature (°C)		*t*	*R*	*P*
H_2_O_2_	20	Morning	8.24	0.992	7.5 × 10^–5^
		Afternoon	5.18	0.891	0.0013
	30	Morning	5.88	0.912	0.00061
		Afternoon	3.38	0.787	0.012
Chl-a	20	Morning	2.81	0.728	0.026
		Afternoon	2.84	0.669	0.048
	30	Morning	4.37	0.856	0.0033
		Afternoon	5.25	0.893	0.0012
Chl-b	20	Morning	2.83	0.730	0.025
		Afternoon	2.39	0.617	0.076
	30	Morning	4.65	0.870	0.0023
		Afternoon	5.97	0.912	0.0056
Car	20	Morning	2.05	0.612	0.080
		Afternoon	3.06	0.757	0.018
	30	Morning	2.71	0.715	0.030
		Afternoon	2.05	0.605	0.084
CAT	20	Morning	4.27	0.850	0.0037
		Afternoon	1.50	0.494	0.176
	30	Morning	11.8	0.976	7.0 × 10^–6^
		Afternoon	1.73	0.547	0.127
POD	20	Morning	7.21	0.943	0.00014
		Afternoon	7.21	0.939	0.00018
	30	Morning	8.97	0.959	4.4 × 10^–6^
		Afternoon	4.26	0.850	0.0037
APX	20	Morning	8.05	0.950	8.8 × 10^–5^
		Afternoon	0.344	−0.129	0.74
	30	Morning	4.13	0.842	0.0044
		Afternoon	2.17	0.635	0.067

*P-values are obtained by one-way analysis of variance (ANOVA) and Bivariate analysis following Pearson’s correlation and t-test.*

**TABLE 6:** *p*-values for the difference between morning and afternoon.

Temperature (°C)	H_2_O_2_	Chl-a	Chl-b	CAR	CAT	POD	APX
20	0.933	0.986	0.654	0.680	0.020	0.695	0.0045
30	0.932	0.443	0.552	0.314	0.018	0.022	0.025

*P-values are obtained by one-way analysis of variance (ANOVA) and Bivariate analysis following Pearson’s correlation, and t-test.*

### Effects of Stress Combination: Light and Iron

*E. densa* also exhibited a highly negative responsive to Fe exposure. Fe is an essential nutrient for plants and is the major metal involved in electron transfer chains, both accepting and donating electrons of photosynthesis and respiration, respectively (Michalak, 2006). However, Fe is toxic when it accumulates to high levels (Michalak, 2006). An excess amount of Fe in a plant leads to an increased formation of ROS (Hell and Stephan, 2003). The antioxidation process, mainly prominent in the tolerant genotype, is achieved by controlling antioxidant enzymes (de Pinto and de Gara, 2004; Halliwell, 2006; Kumar et al., 2014). At higher concentrations, Fe can replace essential metals in pigments and enzymes, disrupting their function; high iron concentrations also reduce the activities of CAT, APX, and other antioxidants (Bielawski and Joy, 1986). In addition to the enzymatic defense, certain amino acids and sulfur metabolites also possess antioxidant properties that reduce ROS damage in Fe-toxic plants, which may reduce the antioxidant enzyme activity (Freeman et al., 2004; Kumar et al., 2014).

In the present study, the Fe toxicity is clearly exhibited. With an increasing Fe concentration in the substrate, the CAT and APX antioxidant levels decreased, which led to an increase in H_2_O_2_ accumulation in the plant tissues. On the other hand, the increased POD activity was proportionate to H_2_O_2_ accumulation, suggesting the low-Fe independent nature of POD. CAT protects cell walls from the destruction caused by H_2_O_2_ production due to iron stress. CAT also plays an important role in the co-degradation of H_2_O_2_ in association with POD (Khan et al., 2018; Figure 8). Due to the inhibiting effect of excess ROS production and damage caused by a massive concentration of free Fe ions, significant decreasing results were observed in CAT, POD, and APX (Khan et al., 2018).

The influence of high Fe content on plants is enhanced with increased light intensity. The plants survived a 10 mg L^–1^ Fe exposure under 30 and 100 μmol m^–2^ s^–1^ PAR intensities, but deteriorated under a 200 μmol m^–2^ s^–1^ PAR intensity, confirming the strong influence of light intensity on the condition of plants.

There is an enhanced correlation between H_2_O_2_ and Fe when controlling the PAR intensity effect, and there are significantly enhanced correlation between H_2_O_2_ and PAR when controlling the Fe effect. This suggests that the accumulation of H_2_O_2_ is caused by both Fe content and high light intensity, independently of the other stressor, in the present experimental range.

The highest accumulation of H_2_O_2_ in the tissues under Fe toxicity and high PAR exposure might exceed the tolerance level—although it is likely species-specific and causes extensive damage to cells (Cheeseman, 2007; Quan et al., 2008; Asaeda et al., 2021).

Many studies have been conducted on the inhibition effect of strong light intensity on photosynthesis (Öquist et al., 1992; Madsen and Sandjensen, 1994; Hanelt, 1998, 1996; Morrissey and Guerinot, 2009). Photoinhibition leads to a decrease in photosynthetic pigments (Chl-a and Chl-b), which is shown in the present results. The presence of H_2_O_2_ in plant tissues is negatively correlated with photosynthetic pigments. However, regardless of the stress source, the Chl-a concentration declines under lower stress intensities than with H_2_O_2_ in the plant tissues. Increasing H_2_O_2_ can lead to Chl-a and Chl-b declines after an 8-hr elevation of *Arabidopsis thaliana* (Rao et al., 1997). The optimum light-harvesting antenna for plants is a Chl a and b ratio of 5 (Wu et al., 2020). However, there was not clear difference in the ratio in the present experiment.

In the present study, the F_v_ F_m_^–1^, which explains the photosystem’s efficiency, proportionately decreased with H_2_O_2_ accumulation rather than Chl-a concentration.

Reduced photosynthesis efficiency negatively influences the prosperity and vigor of plants. This was reflected in the reduced SGR and IAA concentration, which regulated the shoot elongation (Yang et al., 1993; Zhou et al., 2006).

### Possibility of Hydrogen Peroxide Concentration as an Indicator of Environmental Stressors

Chlorophyll fluorescence (F_v_ F_m_^–1^) indicates the efficiency of the photosynthesis rate at PSII, which is highly related to the environmental stress exerted on the plant body. Thus, it is often used to identify the condition of plants. The H_2_O_2_ concentration had a striking negative correlation with the F_v_ F_m_^–1^, regardless of stressor type, in the present study. The exception is the presence of a very high Fe (∼10 mg L^–1^) concentration and high PAR intensity (∼200 μmol m^–2^ s^–1^), under which plants nearly died. There is a similar relationship between H_2_O_2_ and temperature rise in both laboratory and field experiments (Asaeda et al., 2020) in terms of different temperatures and light intensities (Riis et al., 2012). H_2_O_2_ values were slightly higher (∼10 μmol g^–1^FW) with the experiments compared with the field samples, likely because the plants were exposed to radiation only at the tissue’s upper side in the field. Therefore, the difference is considered the result of a possible fluctuation in the field measurements.

The Chl-a and Chl-b concentrations also had a clear unique negative correlation with the H_2_O_2_ concentration in the plant tissue, regardless of stressor types. The type of growth rate parameter, shown by the extension rate, also indicates these unique negative trends. The oxidative stress intensity is based on the activities of H_2_O_2_, as well as other ROS, such as singlet oxygen and hydroxyl radicals, although superoxide is closely related to H_2_O_2_.

However, H_2_O_2_ is the major ROS generated in various organelles; thus, its concentration predominantly implies the level of environmental stress on the plants (Mittler, 2002; Miller and Mittler, 2006; Sharma et al., 2012; Czarnocka and Karpiński, 2018). The present study’s results indicate that H_2_O_2_ concentration has negative but unique correlations with plant growth, photosynthetic pigment content, IAA concentration, and F_v_ F_m_^–1^, regardless of the stressor type. Yet, the H_2_O_2_ response was slightly delayed compared with the photosynthetic pigment concentration.

Plants have opposite trends in their responses to some types of stressors, such as drought and salinity, drought and heat, and drought and high light (Rizhsky et al., 2002; Giraud et al., 2008; Ahmed et al., 2013; Suzuki et al., 2014; Choudhury et al., 2017). However, as for positively interacting stressors, the H_2_O_2_ concentration has the potential to be a good indicator of overall plant condition, at least at a practical management level (Asaeda et al., 2018).

### Photoinhibition of *Egeria densa* in Natural Conditions

The H_2_O_2_ concentration substantially increased in the natural river samples when exposed to high light intensities (Asaeda et al., 2020). Excessive light intensity overloaded electrons generated at PSII. These electrons are transported to PSI, where super oxides are produced from oxygen and then undergo dismutation to H_2_O_2_. They are toxic and damage the PSII protein D1, which otherwise repairs PSII (Leitsch et al., 1994). In this process, photoinhibition is activated more readily under higher light intensities and damages the plant. In the case of submerged plants, normal subjected light intensity is not high; it is several hundred μmol m^–2^s^–1^ PAR at most, which is low compared with that of terrestrial or emergent species (Asaeda and Barnuevo, 2019). At the present study’s sites, the light intensity was approximately 100–200 μmol m^–2^s^–1^ PAR at 0.5 m deep. *E. densa* grew mostly at 0.5–1.0 m deep, and plants grew at depressed sites on the river bottom. Canopy top shoots located less than 10 cm deep were often dying, although the deep shoots were healthy. These results indicate that this species seems to prefer the relatively low light intensity of 100–200 μmol m^–2^s^–1^ PAR to higher light intensities. Thus, exposure to high light intensity could be an efficient method of reducing the community.

## Conclusion

For the management of aquatic plants, growth monitoring after the administration of treatments is usually used; however, this takes a long time to obtain the results. The present study indicates that H_2_O_2_, the most abundant ROS, increases in concentration with the combined stress of high light intensity and Fe concentration, almost independently. Light and Fe stressors originally affect different organelles, but both present similar symptoms of oxidative stress, through H_2_O_2_ generation. With low levels of PAR or Fe, Chl-a and Chl-b negatively correlated with the H_2_O_2_ concentration, while, when exposed to higher levels of stress, the chlorophyll content along with H_2_O_2_ significantly declined.

Increased Fe concentration destroys the activity of CAT and APX, indicating plant tissue damage. Except for 200 μmol m^–2^s^–1^ of PAR, SGR, and IAA also have a negative relationship with Fe concentration. Therefore, with the present results and the previous findings, we suggest that the H_2_O_2_ concentration could be a suitable marker of environmental stress intensity, at least at a practical management level. It also has the potential for monitoring combined stressors if they have positive interaction trends, although more studies are required.

## Data Availability Statement

The raw data supporting the conclusions of this article will be made available by the authors, without undue reservation.

## Author Contributions

TA contributed the conceptualization, filed works, and wrote the manuscript together with other members. MR edited throughout the manuscript. XL made experiments and analysis the manuscript. JS reviewed and commented the manuscript. All authors contributed to the article and approved the submitted version.

## Conflict of Interest

TA was employed by the company Hydro Technology Institute Co, Ltd. The remaining authors declare that the research was conducted in the absence of any commercial or financial relationships that could be construed as a potential conflict of interest.

## Publisher’s Note

All claims expressed in this article are solely those of the authors and do not necessarily represent those of their affiliated organizations, or those of the publisher, the editors and the reviewers. Any product that may be evaluated in this article, or claim that may be made by its manufacturer, is not guaranteed or endorsed by the publisher.
